# Supplementary material for: A small molecule that disrupts S. Typhimurium membrane voltage without cell lysis reduces bacterial colonization of mice
Source: PLoS Pathog. 2022 Jun 10;18(6):e1010606. doi: 10.1371/journal.ppat.1010606 (PMC9223311; doi:10.1371/journal.ppat.1010606)
Supplement: S1 Fig — Fig A. D66 quenches the fluorescent dye DiSC3(5) in a concentration-dependent manner. A) Control wells (without bacterial cells) containing medium with 2 mM DiSC3(5) and DMSO or compound, as indicated, added at time 0. B) Data from Fig 3A normalized to DMSO at time 0 but without correction for the quenching effect of D66 observed in panel A. JD1 was included as a control. Fig B. D66 pharmacokinetic parameters. Values were calculated by compartmental modeling using Phoenix WinNonlin. Data fit a two-compartment model (r = 0.9976) with bolus dosing. A) Decay curve. B) Parameter values. (PDF) [file ppat.1010606.s001.pdf]

S1_Figure
Supporting Information, Dombach et al., PLoS Pathogens, 2022

Figure A in S1 Figure

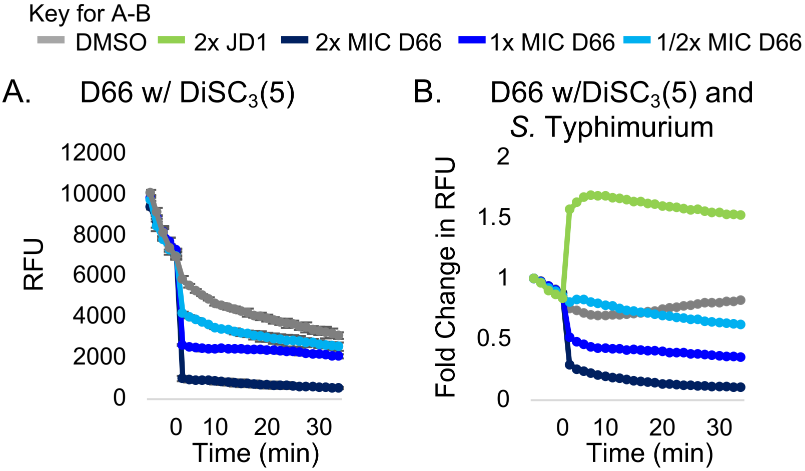

Figure S1. D66 quenches the fluorescent dye $DiSC_3(5)$ in a concentration-dependent manner. A) Control wells (without bacterial cells) containing medium with 2 mM $DiSC_3(5)$ and DMSO or compound, as indicated, added at time 0. B) Data from Fig 3A normalized to DMSO at time 0 but without correction for the quenching effect of D66 observed in panel A. JD1 was included as a control.

Figure B in S1 Figure

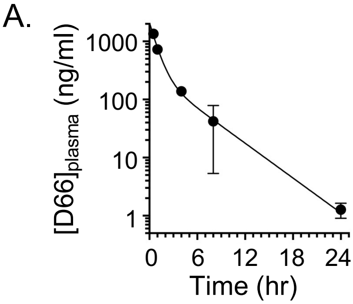

B.

**Parameter**	**Value**
AUC (ng/ml x hr)	2757
C_{max} (ng/ml)	2085
$t_{1/2}$ β (hr)	3.0
V_{ss} (L/kg)	43.3

Figure S2. D66 pharmacokinetic parameters. Values were calculated by compartmental modeling using Phoenix WinNonlin. Data fit a two-compartment model (r= 0.9976) with bolus dosing. A) Decay curve. B) Parameter values.
