# Supplementary material for: A small molecule that disrupts S. Typhimurium membrane voltage without cell lysis reduces bacterial colonization of mice
Source: PLoS Pathog. 2022 Jun 10;18(6):e1010606. doi: 10.1371/journal.ppat.1010606 (PMC9223311; doi:10.1371/journal.ppat.1010606)
Supplement: Attachment — Submitted filename: Response_May06.docx [file ppat.1010606.s002.docx]

**Reviewer's Responses to Questions**

Author’s responses are in blue. We thank all three reviewers for the thoughtful and useful comments. The changes suggested have clearly improved the manuscript.

**Part I - Summary**

**Reviewer #1:** The manuscript by Dombach et al. describes the identification and testing of a small molecule that reduces Salmonella CFU within macrophages and in a mouse model of infection. Attempts to determine the mechanism of action have led them to the bacterial inner membrane, where it disturbs the membrane voltage without permeabilization of the membrane. Mutants with increased resistance to the compound were identified, which mapped to H-NS, but it is not likely the target. Treatment of mammalian cells also showed an effect of the compound on the mitochondrial membrane, where it resulted in hyperpolarization. The identification of a small molecule that affects Salmonella membranes is of interest in the antimicrobial field. However, there are some issues with the data that is presented and the findings reported move this work forward, but not in a significant way. More data is needed.

**Reviewer #2:** Dombach et al. report D66 as a new small molecule that prevents the survival of Gram-negative

pathogen in macrophages. In their manuscript, the authors show that D66 inhibits bacterial growth under specific conditions or in specific genetic backgrounds. They also found that mutations in hns gene confer resistance to D66 in vitro. Finally, they show that D66 can reduce bacterial tissue colonization in mice. The manuscript is original, timely and could interest a large audience. The findings are interesting but additional work is essential to clarify the current model. These experiments could severely change the interpretation of the manuscript and the current discussion.

**Reviewer #3:** The authors use a screening platorm based on tissue culture cell infection assay to identify compounds that specifically target S. Typhinurium in macrophages. Here the authors charachterize D66, a small molecule with anti S. Typhinurium activity in macrophages. They find that macrophage mediated damage ot the cell envelope facilitates D66 antimicrobial activity, but D66 itself is not an outer membrane permeabilizing agent. Instead, once D66 is capable of getting through the OM, it then perturbs the voltage across the cytoplasmic membrane without permeabilizing it. Despite hyperpolarization of mitochondrial membranes in response to D66, infected macrophages survived better with D66 treatment, presumably down to the anti-S. Typhinurium effects. Furthermore, D66 showed efficacy in a mouse model of infection, without associated toxicity. Overall, this is a well thought out study. The data is clear and appropriately interpreted.

**Part II – Major Issues: Key Experiments Required for Acceptance**

**Reviewer #1:**

1. The primary message of this manuscript, that the cytoplasmic membrane is a good antimicrobial target, was the same message as these authors’ previous PLoS Pathogens manuscript on JD1.

Yes, part of the message is that the cytoplasmic membrane is a good antimicrobial target. The other part is that the compound presented, D66, lacks membrane damaging effects, as indicated in the title (“without cell lysis”) and in the last sentence of the abstract (“These data suggest that the inner membrane could be a viable target for anti-Gram-negative antimicrobials, and that disruption of bacterial membrane voltage without lysis is sufficient to enable clearance from the host”).

2. Was D66 identified in the screen in which they identified JD1? This is not clear from the manuscript. Why was this compound chosen for study?

We added the underlined text to (L125), “This compound, which has not been previously studied, was found to be highly active in a high content screening platform known as SAFIRE (Screen for Anti-infectives using Fluorescence microscopy of IntracellulaR Enterobacteriaceae) (19).”

3. The authors should discuss why they think the compound works in macrophages but not epithelial cells.

L412: We added a short paragraph to the discussion on this topic.

4. The authors should focus just on D66. They occasionally bring a previously studied compound JD1 into the

discussion. This seems a bit distracting at times.

We have limited text on JD1 to experiments in which it was used as a control for comparison to D66.

5. Figure 1D. It is unclear why there are low IC50 ratios described when this data told the authors that there is no effect of the compound on HeLa cells.

L136: We expanded on the description of the HeLa cell data with the underlined text, “Since *S.* Typhimurium can replicate in other cell types, we also tested the ability of D66 to reduce bacterial load in HeLa cells, which are derived from epithelial cells. The compound had little effect; while the calculated IC_50_s are low, the 5-10-fold reduction in CFU, compared to the >1000-fold CFU reduction in macrophages does not give confidence that D66 is highly active in HeLa cells (Fig 1D).”

6. Figure 2A and B. Why didn’t the authors do a typical growth curve, and instead use stationary phase bacteria? A typical growth curve examining D66 effects should be performed like in 3C- F. Also, it is unclear why there are the blank spaces to the left of the data in each panel. Finally, why didn’t the authors use polymyxin alone as a control? This needs to be included as you cannot just rely on the literature source for the concentration that is sub-lethal.

The dose response curves were performed with overnight stationary phase cells diluted to an OD of 0.01 in fresh media and then monitored for absorbance over 18 hours. This is a standard approach for calculating the effective concentration of a compound across different conditions and enables comparison to the SAFIRE and CFU X-axes in Figure 1 B-D. In Figure 2 A and B, we minimized the blank space on the left side of the graphs as much as possible while retaining the same X-axes. The PMB controls in Figure 2 A and B are the red squares. The literature source cited for determination of a sub-inhibitory PMB concentration is a recent paper from our lab, which we now state in the text.

7. Figure 2C and D. The checkerboard assays are unclear. It appears there is only activity at the highest concentration of novobiocin. There are some sporadic changing of the colors in panel C. Why is this? The authors should try to perform a fractional inhibitory concentration index from the checkerboard data. This can identify synergy, additivity, etc.. The authors stated that there was synergy online 170, but there is no data affirm that. Finally, a control of a compound that does affect the outer membrane (like polymyxin B) would have been useful as a control in this figure.

We cleaned up the checkerboards by changing the color scale from 48 – 100 % to 0 – 100 %, which reduced the appearance of “sporadic” color changes. We also added a PMB-novobiocin checkerboard as a control as suggested (Fig 2C). Since calculation of FICIs requires a concentration at which D66 inhibits growth, which does not exist, we followed the implied suggestion that we be more careful with use of the word “synergy” and have in this section changed it to “potentiation”.

8. In the in vitro evolution experiments, the authors should have used two or more independent colonies, as just performing the evolution with one colony can lead to siblings from an early mutational event. In fact, you did have four of the sequenced mutants that were identical. Also, for figure 4 and this data, the authors should determine the sensitivity of the evolved strains to D66 in comparison to the parent strain. Finally, putting the H-NS mutations into a tolC mutant background and the WT strain is warranted to determine if D66 resistance mutations in H-NS are only effective in the acrAB mutant background.

The reviewer misunderstands how the *in vitro* evolution experiments were performed, and in response we added clarifying text to the results and to the methods. Briefly, a single clone/colony was distributed into eight liquid samples, six that were treated with D66 and two that were vehicle-treated. All eight samples were sequenced, and the vehicle-treated samples revealed vehicle-dependent changes. This is a cleaner way of selecting for and analyzing resistant mutants than picking and evolving single colonies, which likely have different background polymorphisms. The acquired polymorphisms in the resistant strains with *hns* mutations were not the same, indicating independent evolution over the course of the experiment.

Construction and examination of *tolC* mutations in the *hns* mutant resistant strains is not likely to provide significant new information about D66 because loss-of-function mutations in *hns* increase the expression of multiple TolC-dependent efflux pumps (Cho and Misra, 2021). Nevertheless, the reviewer is correct in that it would be good in future manuscripts to explore whether each of the efflux pumps induced in *hns* mutants export D66 and/or derivatives of D66.

Cho HJ, Misra R. Mutational Activation of Antibiotic Resistant Mechanisms in the Absence of Major Drug Efflux Systems of Escherichia coli. J Bacteriol. 2021 May 10.

9. Figure 5. In panel A, the authors state that the compound D66 has a dose dependent hyperpolarization effect. That is not true from the graph as the 56 umolar concentration shows much less polarization than the lower concentrations, which escalate with increasing dose.

L 313: We added that at the highest concentration of D66, hyperpolarization increases and then declines with time towards that of solvent-treated control, possibly due to compound aggregation and clearance.

10. Figure 5. Examination of infected cells in the LDH assay complicates the scenario as infection causes LDH release. It looks clear from the uninfected that there is no LDH release. I’d just present that.

L321: We have clarified the importance of examining compound activity in both infected and uninfected cells: their biology differs, and the data confirm that D66 inhibits infection.

11. In the animal experiments, the PK data was achieved with one dose of D66 but the effect on Salmonella infected mice was performed with two doses of D66. These are not comparable. Also, in line 320, the authors’ state that the compound was tolerated in vivo but that is only over a 48 hour period after which they sacrifice the mice. This should be stated with the caveat that mice were only examined after 2 days, where pathology might not even have a chance to develop.

The PK data were obtained from mice treated with a single dose of D66, the same dose used in the infected animals, as indicated. If a derivative of D66 becomes a lead compound, it would be appropriate to do more extensive PK testing, for instance, with two doses, with the lead compound.

L332, L349: The text clearly states that the analysis of toxicity was only for 24 hours and that in the infection experiment, “All mice that received D66 survived in good condition out to 48 hours”.

12. Did the authors consider using efflux pump poisoning agents to see if that increased the activity of their compounds?

This is a good idea and will be considered for future work, for instance to confirm that a compound is an efflux pump substrate. Since most efflux pump inhibitors appear to target multiple pumps, significant genetic analyses will be needed to interpret results.

**Reviewer #2**:

-Line 132 - “D66 inhibits bacterial growth under conditions that compromise the cell envelope”. The

data of this section suggest that D66 is pumped out by AcrAB efflux pump (as suggested by the authors on line 231). Stresses on the cell envelope could affect AcrAB activity. Authors should test the activity of AcrAB in presence of PMB (described here: PMID: 27381291; Hoecth or PI assay). In addition, it is well known that PMB is a substrate of the AcrAB efflux machinery in enterobacteria. Therefore, PMB could sensitize cells to D66 by directly competing for the same efflux machinery. Finally, it is unclear if it is D66 or the PMB itself that affects bacterial physiology in these conditions. The authors should test the effect of PMB on the acrAB mutant, as done on panels 2A and B.

L386: We appreciate these points and now explain in the discussion that competition between PMB and D66 for efflux pumps could play a role but is not likely. While PMB is indeed exported by AcrAB from *Klebsiella pneumoniae,* in *E. coli* and *S.* Typhimurium*,* LPS modification confers resistance to low to moderate concentrations of PMB. At the high concentrations of PMB that overcome LPS modifications, MtdEF appears to be a more likely PMB exporter than AcrAB. In addition, we tested the effects of PMB on the *acrAB* mutant in panels 2A, B and observed a leftward shift, as expected.

-The importance of AcrAB suggests that efflux pumps limit the entry of the D66 inside the cells. Since D66 is

presented as an alternative to traditional antibiotics, authors should check if mutations in AcrAB that confers

antibiotic resistance (e.g. fluoroquinolones) protect Salmonella against the D66 compound during infection.

These kinds of experiments are important and would be most appropriate to include in a manuscript that attempts to develop derivatives of D66 into a lead compound. Interpretation will be complicated by the large periplasmic substrate binding pocket of AcrB and prior observations that substrates do not always bind in the same part(s) of the pocket.

- Line 176 – In this section, the authors used PI to assess membrane permeabilization during D66 treatment.

However, since PI labeling is also dependent on the activity of AcrAB, these results could be misinterpreted. One hypothesis could be that D66 promotes PI labeling by competing for the same efflux machinery. Please clarify this section.

This is an interesting idea, that D66 and PI compete for AcrB. We could not find the corresponding reference by searching on, for example, “Acr” and “propidium iodide”. We did find a 2014 manuscript that states “…propidium iodide, which is not a known AcrB substrate.”

ACS Synth Biol. 2014 Jan 17;3(1):30-40. doi: 10.1021/sb400065q. Epub 2013 Sep 13.

Enhancing tolerance to short-chain alcohols by engineering the Escherichia coli AcrB efflux pump to secrete the non-native substrate n-butanol [Michael A Fisher](about:blank)  [^1^](about:blank#affiliation-1) , [Sergey Boyarskiy](about:blank), [Masaki R Yamada](about:blank), Niwen Kong, [Stefan Bauer](about:blank), [Danielle Tullman-Ercek](about:blank)

- According to their data, the simplest model is that D66 is directly targeting Hns. Did the authors test this

hypothesis? Mutations in hns could limit the interaction between the drug and Hns itself. These data are extremely interesting but are unclear and not sufficiently addressed in the manuscript. The authors suggest in their discussion that loss of Hns could promote the expression of alternative efflux pumps. The authors should perform efflux assay (with PI or Hoecht) with their Hns mutants to validate their hypothesis.

At face value, we agree that the simplest model is that D66 directly targets H-NS. However, Cho and Misra 2021 recently showed that loss-of-function *hns* mutations increase the expression of alternative efflux pumps, suggesting that D66 is instead exported by one or more of these pumps. We now explain the Cho and Misra results more fully, as was needed.

**Reviewer #3:** The search for for resistant mutants is performed in a mutant that already has some compromised fitness, in order to create in vitro sensitivty. The mutations selected for in this mutant background may be synthetically lethal to the bacteria in macrophages while easily maintained in a wt background in macrophages. The selection of resistant mutants should be performed in the presence of sub-inhibitory PMB as in Figure 2. That way, you generate resistance directly in the envelope compromising conditions, similar to those experienced in macrophages and then test that mutant for fitness defects in macs. It would be a far cleaner way to determine if resistant mutants, retaining fitness, are quickly selected for by D66 treatment.

As requested, we selected for resistant mutants in the presence of sub-inhibitory concentrations of PMB and added this data to the results (Fig 4C). We obtained 1 tolerant isolate and 5 resistant clones. The latter had increased resistance to PMB alone. These data strengthen the manuscript by suggesting that a combination of the outer membrane and efflux pumps normally keep D66 out of the cell. They also corroborate prior observations from other laboratories showing that it is fairly easy to obtain mutations that confer resistance to PMB, as multiple genes regulate this process, as now explained in the text.

**Part III – Minor Issues: Editorial and Data Presentation Modifications**

**Reviewer #1:**

The points below are appreciated and changes have been made to accommodate each suggestion.

13. Line 54. “..relatively impervious to treatment” is too broad of a statement.

14. Line 57 to 58. This sentence is out of place in the author summary.

15. Line 72. Perhaps change “in the periplasm” to “from the periplasm”

16. Line 75. Change the sentence to “…(e.g. serum) and the contents of phagolysosome after phagocytosis”.

17. Line 78. Move “during infection” to the front of the sentence and remove the word “thus”

18. Line 293- 294. There are many other differences between bacterial and mammalian cell membranes than charge.

19. Line 410 through 411. The D66 statement is not a sentence.

20. Line 424. Please fix the spelling of gentamicin

**Reviewer #2:**

- Line 67 – “The negative charges on LPS are stabilized by recruited cations, creating a coat around the bacterium that excludes many antibiotics”. This sentence is a bit counterintuitive since antibiotics are effective against bacteria. This sentence suggests that LPS allows bacteria to grow in presence of antibiotics by excluding them (antibiotic resistance). Please clarify.

L 74. We added the underlined words to complete the thought: “The negative charges on LPS are stabilized by recruited cations, creating a coat around the bacterium that excludes many antibiotics effective against Gram positive bacteria”

- Table1 - Define IC50, MIC50, and cMIC95 in the legend of the table

These definitions were needed and have been added.

- Line 166 – The rationale used by the authors in this section is unclear. The sensitivity of the acrAB mutant suggests that D66 needs to enter the cell to mediate its effect. Therefore, the study of the outer membrane seems counterintuitive. Please clarify this point in this section and in the discussion.

L178 – We agree, the addition of the reviewer-suggested rationale strengthens this section.

**Reviewer #3:** Although the data support D66 not damaging the outer membrane, it would make more sense to

include polymyxin B nonapeptide as a positive control in the novobiocin synergy assay in Figure 2. Here, you expect a 100-fold increase in sensitivity to novobiocin, which would confirm the appropriateness of the experimental procedures in ruling in, or out, OM damage.

We agree that a positive control was needed for novobiocin potentiation and have added a PMB-novobiocin checkerboard (Fig 2C).
